# Impact of neonatal hyperbilirubinemia on peripheral neuromuscular development: evidence from muscle fiber conduction velocity measurements

**DOI:** 10.3389/fped.2025.1691272

**Published:** 2026-01-06

**Authors:** Kun Wang, Wei Zhao, Ling Song, Yeying Feng, Qiuyue Kou, Tieyan Wang

**Affiliations:** Department of Neonatology, The Second Affiliated Hospital of Qiqihar Medical University, Qiqihar, Heilongjiang, China

**Keywords:** innervation zone, motor unit activity, muscle fiber conduction velocity, neonatal hyperbilirubinemia, peripheral neuromuscular development, surface electromyography

## Abstract

**Background:**

Neonatal hyperbilirubinemia is a common condition that may impair neurodevelopment, yet its impact on peripheral neuromuscular function remains underexplored.

**Objective:**

This study aimed to assess the effects of hyperbilirubinemia on muscle fiber conduction velocity (MFCV) in neonates using surface electromyography (sEMG).

**Methods:**

MFCV was estimated from tibialis anterior sEMG recordings during passive and isometric contractions in neonates with and without hyperbilirubinemia. Global and local time-delay strategies were applied. Z-score analysis and repeated-measures ANOVA were used to compare groups, while regression analysis examined MFCV temporal trends.

**Results:**

The hyperbilirubinemia group exhibited significantly lower MFCV and Z-score values than controls (*p* < 0.001). Control infants showed characteristic spatial and temporal MFCV patterns, including arch-shaped conduction profiles and time-dependent declines, which were absent in the hyperbilirubinemia group. Disorganized innervation zone (IZ) distributions and reduced conduction variability further indicated impaired neuromuscular development.

**Conclusions:**

Neonatal hyperbilirubinemia may alter peripheral neuromuscular maturation. sEMG-based MFCV estimation may serve as a potential sensitive and noninvasive electrophysiological biomarker for detecting bilirubin-related neuromuscular impairment in early infancy.

## 1 Introduction

Neonatal hyperbilirubinemia (NHB) is one of the most common clinical conditions in the early postnatal period, affecting more than half of term infants and the majority of preterm neonates (1, 2). Although most cases are benign and self-limiting, excessively elevated bilirubin levels can lead to acute bilirubin encephalopathy, resulting in adverse neurobehavioral and neurodevelopmental outcomes (1–3). In clinical practice, neonates with bilirubin levels exceeding treatment thresholds are typically managed with phototherapy or exchange transfusion, and total serum bilirubin (TSB) levels often return to normal shortly after intervention. However, accumulating evidence suggests that even in the absence of clinically diagnosed acute bilirubin encephalopathy, or after bilirubin levels have normalized, early exposure to high bilirubin may still exert detrimental effects on the developing nervous system (2, 4, 5). Some infants may later develop severe neuromuscular abnormalities, such as quadriplegic cerebral palsy, choreoathetosis, and/or dystonia (6, 7). These conditions typically cannot be definitively diagnosed until 15 months of age or later (1, 6). Therefore, early assessment of peripheral neuromuscular function in neonates with hyperbilirubinemia is of great importance for understanding long-term outcomes.

Electromyography (EMG) is a key clinical tool for assessing peripheral neuromuscular function and has been widely applied to detect neuromuscular alterations (8–12). With advances in EMG acquisition technology, surface electromyography (sEMG), due to its non-invasive nature, has gained broad acceptance in pediatric populations (13–15). In recent years, numerous studies have demonstrated that abnormalities in sEMG features (such as morphology, amplitude, spatiotemporal patterns, frequency, and coherence, etc.) can effectively quantify neuromuscular dysfunction in neonates. For instance, Xiong et al. (16) proposed that abnormal intermuscular coherence parameters may serve as potential biomarkers for assessing neonatal neurodevelopmental responses. Dolinskaya et al. (17) employed an event-based time-domain sEMG analysis approach to reveal notable differences in the developmental patterns of muscle activation in preterm infants. Furthermore, Siegel et al. (18) applied amplitude-based sEMG analysis to evaluate postural control in newborns, providing a quantitative foundation for early assessment of motor function. While these methods show promise in characterizing neonatal muscle strength and motor unit (MU) recruitment, they may lack sufficient sensitivity to capture bilirubin-related developmental abnormalities (1, 3–5).

Muscle fiber conduction velocity (MFCV) is a key electrophysiological parameter reflecting the integrity of neuromuscular transmission (19–22). It represents the speed at which action potentials propagate along muscle fibers and is influenced by multiple factors, including membrane excitability, fiber diameter, degree of myelination, and metabolic state (20, 23, 24). In neonates, the neuromuscular system is still undergoing rapid development, and any disruption (such as hypoxia, inflammation, or bilirubin toxicity) may result in altered conduction velocity (2). While the neurotoxic effects of hyperbilirubinemia on the central nervous system, such as kernicterus, have been extensively studied, its impact on peripheral motor pathways and muscle conduction function has received relatively little attention (3, 25). Emerging evidence suggests that the effects of hyperbilirubinemia may not be confined to the central nervous system. *In vitro* studies and animal models have shown that bilirubin can reduce peripheral nerve excitability, impair synaptic efficiency, and alter muscle membrane potentials (26–28). These changes, in theory, may manifest as a measurable reduction in MFCV in affected neonates, offering a potential electrophysiological marker of neuromuscular development. However, there is a lack of systematic studies directly examining the relationship between TSB levels and MFCV in human neonates.

This study aimed to investigate the effect of hyperbilirubinemia on MFCV in neonates using sEMG. MFCV was compared between neonates with elevated bilirubin levels and healthy controls by recording sEMG signals from the tibialis anterior muscle during passive concentric contractions. By employing an sEMG-based approach to estimate MFCV, this study offers a novel perspective for evaluating bilirubin-induced changes in neuromuscular conduction. Establishing this association may help elucidate the peripheral manifestations of bilirubin neurotoxicity and support the use of MFCV as a noninvasive biomarker for the early detection of bilirubin-related neuromuscular impairment in neonates. These findings may provide new insights into the neuromuscular developmental consequences of hyperbilirubinemia and contribute to the development of targeted early intervention strategies in at-risk neonates.

## 2 Methods

### 2.1 Dataset description

#### 2.1.1 Subjects

A total of 15 neonates diagnosed with hyperbilirubinemia (8 females and 7 males; gestational age: 40 ± 1.2 weeks) and 15 age- and gender-matched healthy controls (8 females and 7 males; gestational age: 40 ± 1.2 weeks) were enrolled in this study. To ensure adequate statistical power, an *a priori* power analysis was performed using G*Power (version 3.1), applying a two-sided independent-samples t-test (*α* = 0.05, powe*r* = 0.80) to calculate effect sizes and justify the sample size. All neonates in the hyperbilirubinemia group exhibited TSB levels exceeding 18 mg/dL within the first week after birth. None of the participants exhibited clinical signs or had a family history of neuromuscular disorders. To minimize confounding factors that might independently influence neuromuscular development, medical records were examined by two experienced neonatologists. Infants with gestational age < 37 weeks, congenital malformations, metabolic disease, perinatal asphyxia (defined as 1-minute and/or 5-minute Apgar score ≤8, need for endotracheal resuscitation, or umbilical arterial pH < 7.00), or suspected/confirmed neonatal sepsis were excluded. Neonatal sepsis was ruled out based on the absence of positive blood cultures and abnormal inflammatory markers, including C-reactive protein (CRP) ≥ 10 mg/L or procalcitonin (PCT) ≥ 2 ng/mL, as well as no requirement for empirical antibiotic therapy for ≥5 days. Importantly, all enrolled neonates demonstrated 1-minute and/or 5-minute Apgar scores > 7 and stable vital signs, and none required prolonged respiratory support after birth. No participant had a family history or clinical signs of neuromuscular disorders, ensuring that the observed electrophysiological findings were not attributable to prematurity, asphyxia, sepsis, or acute neurological injury but were instead associated with neonatal hyperbilirubinemia. The study protocol was approved by the Clinical Medicine Research Ethics Committee of the Second Affiliated Hospital of Qiqihar Medical University (QMU, Qiqihar, Heilongjiang 161006, China). Written informed consent was obtained from the parents or legal guardians of all enrolled neonates. Table 1 displays the information of all subjects.

**Table 1:** Demographic information of subjects in the NHB and control groups, respectively.

Group	Subject ID	Sex	Gestational age (wk)	Birth weight (Kg)	Delivery mode	Apgar score 1-minute	Apgar score 5-minute	Maximum bilirubin Level (mg/dL)	Phototherapy
NHB	1	F	37	2.53	CS	8	10	19.4	Y
	2	M	41	3.32	VD	10	10	18.1	N
	3	M	38	3.10	CS	9	10	21.6	N
	4	F	38	3.29	VD	10	10	22.3	N
	5	M	39	3.50	CS	10	10	18.5	Y
	6	M	39	3.25	CS	10	10	19.2	N
	7	F	39	3.00	CS	10	10	20.3	N
	8	M	40	3.33	VD	9	10	21.5	N
	9	F	40	3.17	CS	9	10	22.9	Y
	10	M	40	3.32	VD	10	10	19.8	N
	11	M	40	3.24	VD	10	10	19.1	N
	12	F	40	3.08	VD	10	10	21.3	Y
	13	F	40	3.23	VD	9	10	23.8	N
	14	F	41	3.15	VD	10	10	19.1	N
	15	F	41	3.80	CS	10	10	18.8	Y
	1	F	37	2.98	VD	10	10	11.8	N
	2	M	41	2.87	VD	10	10	10.2	N
	3	M	38	3.03	CS	10	10	8.1	N
	4	F	38	3.50	VD	10	10	9.1	N
	5	M	39	3.75	VD	10	10	11.3	N
	6	M	39	3.25	VD	10	10	9.5	N
Control	7	F	39	3.01	CS	10	10	8.9	N
	8	M	40	3.20	VD	10	10	10.7	N
	9	F	40	3.40	VD	10	10	9.3	N
	10	M	40	3.21	VD	10	10	13.3	N
	11	M	40	3.53	VD	10	10	12.1	N
	12	F	40	3.18	VD	10	10	12.5	N
	13	F	40	3.25	VD	10	10	8.7	N
	14	F	41	3.14	CS	10	10	11.6	N
	15	F	41	3.60	CS	10	10	12.4	N

M, male; F, female; VD, vaginal delivery; CS, cesarean section; Y, received phototherapy;, did not receive phototherapy; All enrolled participants did not require NICU admission or exchange transfusion.

#### 2.2.2 Experiment

**Figure 1:**
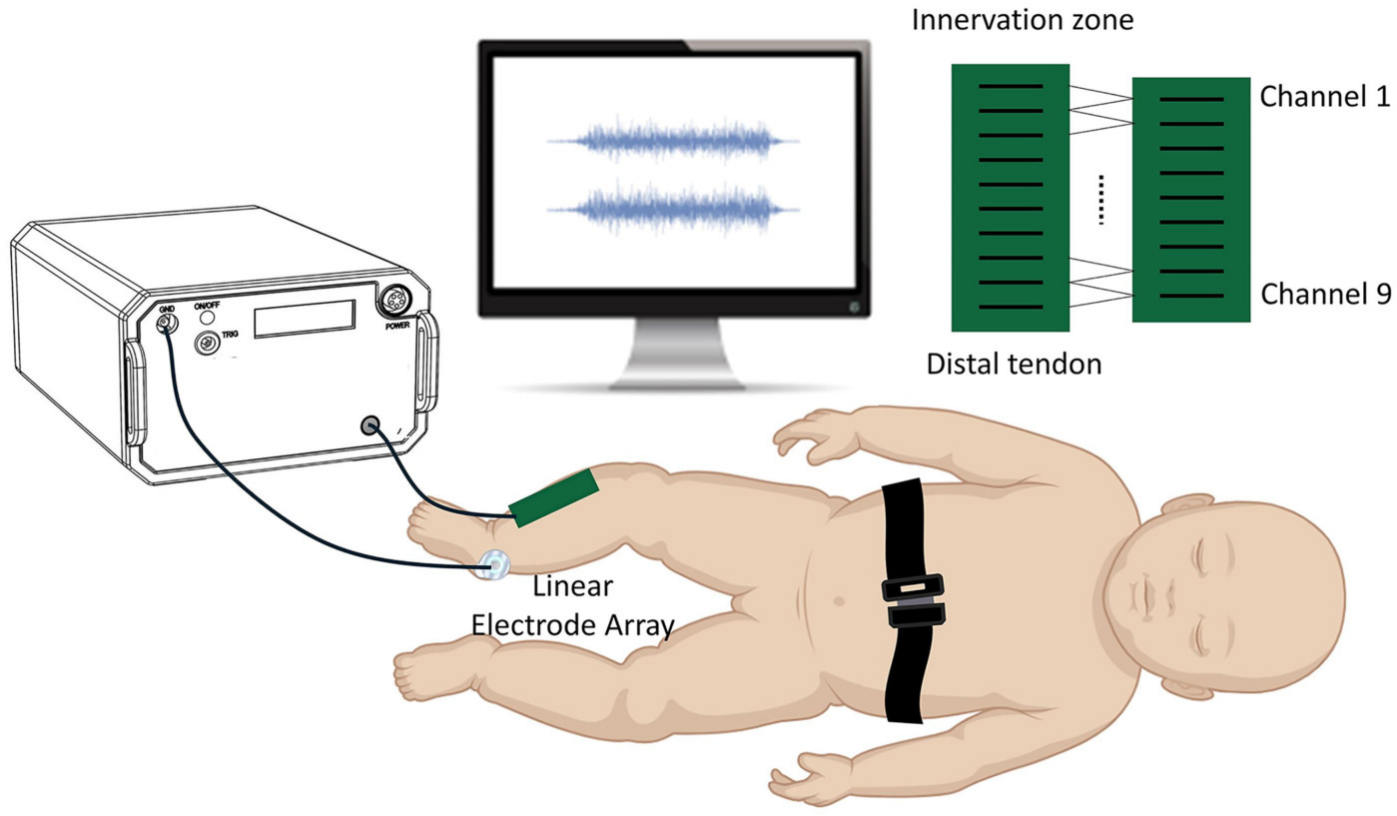
Experimental setup and linear electrode array used for assessing MFCV in the tibialis anterior of neonates.

At six months post-discharge, each neonate underwent MFCV assessment. During the experiment, the neonate was placed in a standard supine position on a custom-designed positioning pad, with the head maintained in a neutral position and the limbs naturally extended. To minimize movement artifacts, the torso was gently stabilized using an adjustable belt integrated into the pad. After cleansing the skin over the tibialis anterior muscle with 70% medical alcohol, a custom-designed linear electrode array was carefully positioned parallel to the muscle fibers, between the innervation zone and the distal tendon. The electrode array consisted of 10 silver bars (each 10 mm in length and 1 mm in width), evenly spaced at 5 mm intervals (see Figure 1), and was secured to the skin. The electrode array was affixed to the skin using an elastic band. A reference electrode was placed over the ipsilateral medial malleolus. Prior to electrode placement, the innervation zone was identified by visually inspecting multi-channel EMG signals to locate the reversal point of motor unit action potential (MUAP) propagation. The innervation zone and the distal tendon were then marked on the skin. The sEMG signals were monitored in real time throughout both the setup and recording phases to ensure signal quality and accurate placement. During the EMG acquisition session, each infant also underwent a brief clinical neuromotor assessment performed by a licensed pediatric physical therapist. Muscle tone was evaluated through passive limb movement and resistance testing, with no signs of hypertonia, hypotonia, or asymmetrical muscle response. Spontaneous movement patterns—including kicking, reaching, and anti-gravity limb activity—were visually inspected to assess symmetry, smoothness, and age-appropriate motor behavior. In addition, a simplified version of the motor domain of the Denver Developmental Screening Test II was administered to evaluate age-referenced motor developmental milestones, such as head control, spontaneous grasping, and limb coordination. All infants exhibited motor behaviors appropriate for their corrected age, and no clinically evident signs of developmental delay or neuromotor abnormalities were observed during the assessment.

Passive ankle plantarflexion movements were performed by a licensed pediatric physical therapist at a constant angular velocity of approximately 10 °/s. An angle sensor was used to monitor joint motion in real time to ensure smooth and consistent movement, although the angle data were not included in the subsequent analysis. During each trial, the infant was guided to produce a stable isometric contraction lasting 3–5 s. Five trials exhibiting consistent and clearly identifiable sEMG responses during the isometric phase were selected for analysis. All recordings were obtained from the right side of the body for both hyperbilirubinemic and healthy control infants. The sEMG signals were acquired using the Porti EMG system (TMS International, The Netherlands) at a sampling rate of 2,000 Hz. The raw data were exported to MATLAB (version R2015a, MathWorks, Natick, MA, USA) for offline processing and analysis.

#### 2.2.3 Data preprocessing and segmentation

For each trial, the raw sEMG signals were first visually inspected to identify a stable 3-second segment. A band-pass filter (20–500 Hz) was applied to attenuate low-frequency motion artifacts and high-frequency noise, and a 50 Hz notch filter was used to suppress power line interference. Next, differential processing was performed by calculating the successive differences between the 10 monopolar channels, yielding 9 bipolar channels. From each trial, a 3-second segment (corresponding to 6,000 samples at a 2 kHz sampling rate) was extracted. This segment was then divided into 12 non-overlapping time windows, each 250 ms in length (500 samples), to ensure adequate temporal resolution for capturing MUAPs in subsequent conduction velocity analysis.

#### 2.2.4 MFCV analysis of sEMG

The MFCV was estimated from the 9 bipolar sEMG time series. Specifically, MFCV was defined and calculated as shown in Equation 1:

$$\mathrm{MFCV}=\frac{d}{\tau }$$ (1)

where *d* represents the inter-electrode distance (i.e., spatial interval between bipolar channels), and *τ* denotes the time delay of signal propagation between channels. Two cross-correlation–based strategies were employed to estimate *τ*, namely the global scheme and the local scheme.

In the global scheme, for each pair of bipolar signals *x*(*t*) and *y*(*t*) within a signal segment, the time delay τc was estimated by identifying the lag corresponding to the peak of the cross-correlation function computed over the entire segment. The final global time delay ${\hat{\tau }}_{\mathrm{global}}$ for the segment was calculated by averaging the delays from *C* − 1 equidistant channel pairs (out of *C* total bipolar channels). The global MFCV estimate for the segment, denoted as MFCV*_global_*, was then derived as shown in Equations 2–5:

$$R_{xy}(\tau )=\sum_{n=0}^{N-1}x[n]\cdot y[n+\tau ]$$ (2)

$${\hat{\tau }}_{c}=\arg\;\max R_{xy}(\tau )$$ (3)

$$\tau_{\mathrm{global}}=\frac{1}{C-1}\sum_{c=1}^{C-1}{\hat{\tau }}_{c}$$ (4)

$$\mathrm{MFC}V_{\mathrm{global}}=\frac{d\cdot f_{s}}{\tau_{\mathrm{global}}}$$ (5)

where *d* is the inter-electrode distance and *fs* the sampling frequency.

The local scheme was designed to characterize temporal variations in conduction velocity during sustained muscle contractions by applying a windowed cross-correlation approach. For each *k*-th time window [*k*S, *k*S + *L* − 1], the time delay ${\hat{\tau }}_{k}$ was estimated as shown in Equations 6–7:

$$R_{xy}^{k}(\tau )=\sum_{n=ks}^{kS+L-1}x[n]\cdot y[n+\tau ]$$ (6)

$${\hat{\tau }}_{k}=\arg\;\max R_{xy}^{k}(\tau )$$ (7)

The MFCV within window *k*, denoted as MFCV*_k_*, was then computed by Equation 8:

$${\mathrm{MFCV}}_{k}=\frac{d\cdot fs}{{\hat{\tau }}_{k}}$$ (8)

For the local scheme, in addition to analyzing the temporal evolution of MFCV within each trial, an overall MFCV estimate was computed to summarize local conduction characteristics. Specifically, the average time delay across all *K* windows in a given trial was calculated as shown in Equation 9:

$$\tau_{\mathrm{local}}=\frac{1}{K}\sum_{k=1}^{K}{\hat{\tau }}_{k}$$ (9)

The corresponding local muscle fiber conduction velocity was then derived as shown in Equation 10:

$$\mathrm{MFC}V_{\mathrm{local}}=\frac{d\cdot fs}{\tau_{\mathrm{local}}}$$ (10)

This estimate reflects the average propagation speed of MUAPs across the entire contraction period, while still preserving temporal sensitivity within the windowed structure. For each participant, both global and local muscle fiber conduction velocity MFCV_global_ and MFCV_local_, were computed by averaging across all valid trials. These participant-wise averages were denoted as M*_g_* and M*_l_*, respectively.

To assess neuromuscular changes in neonates with hyperbilirubinemia, a Z-score based evaluation was performed. Specifically, the Z-scores for each hyperbilirubinemic neonate under the global and local schemes were defined as (M*_g_* − μ_c_)/σ_c_ and (M*_l_* − μ_c_)/*σ*_c_, where μ_c_ and *σ*_c_ representing the mean and standard deviation of MFCV_global_, or MFCV_local_ across all healthy control subjects. Based on criteria proposed in prior literature (29, 30), a Z-score within the range of ±2.5 was considered normal, whereas values outside this range were classified as indicative of neuromuscular changes.

#### 2.4.5 Evaluation of the MFCV–time relation

For both the hyperbilirubinemic neonates and the control group, linear least-squares regression was performed on the average MFCV values across all time windows. The slope and the coefficient of determination (R^2^) were calculated to quantify the rate of change and the goodness of fit, respectively.

#### 2.2.6 Statistical analysis

To verify group differences, one-way repeated-measures ANOVA was performed on both Z-score and MFCV values, with subject group (two levels: NHB and control) treated as the between-subjects factor. Prior to each analysis, the homogeneity of variances was assessed using an *F*-test for both Z-score and MFCV data. To evaluate the relationships of MFCV with both bilirubin levels and time, linear regression was conducted across different time segments of muscle contraction to calculate the slope of the MFCV–time and MFCV–bilirubin relationships. The resulting slope values were then subjected to one-sample *t*-tests to determine whether they significantly differed from zero. The level of statistical significance was set at *p* < 0.05 for all analyses. When necessary, *post hoc* pairwise comparisons were conducted using the Bonferroni correction. All statistical analyses were performed using SPSS software (version 16.0; SPSS Inc., Chicago, IL, USA).

## 3 Results

**Figure 2:**
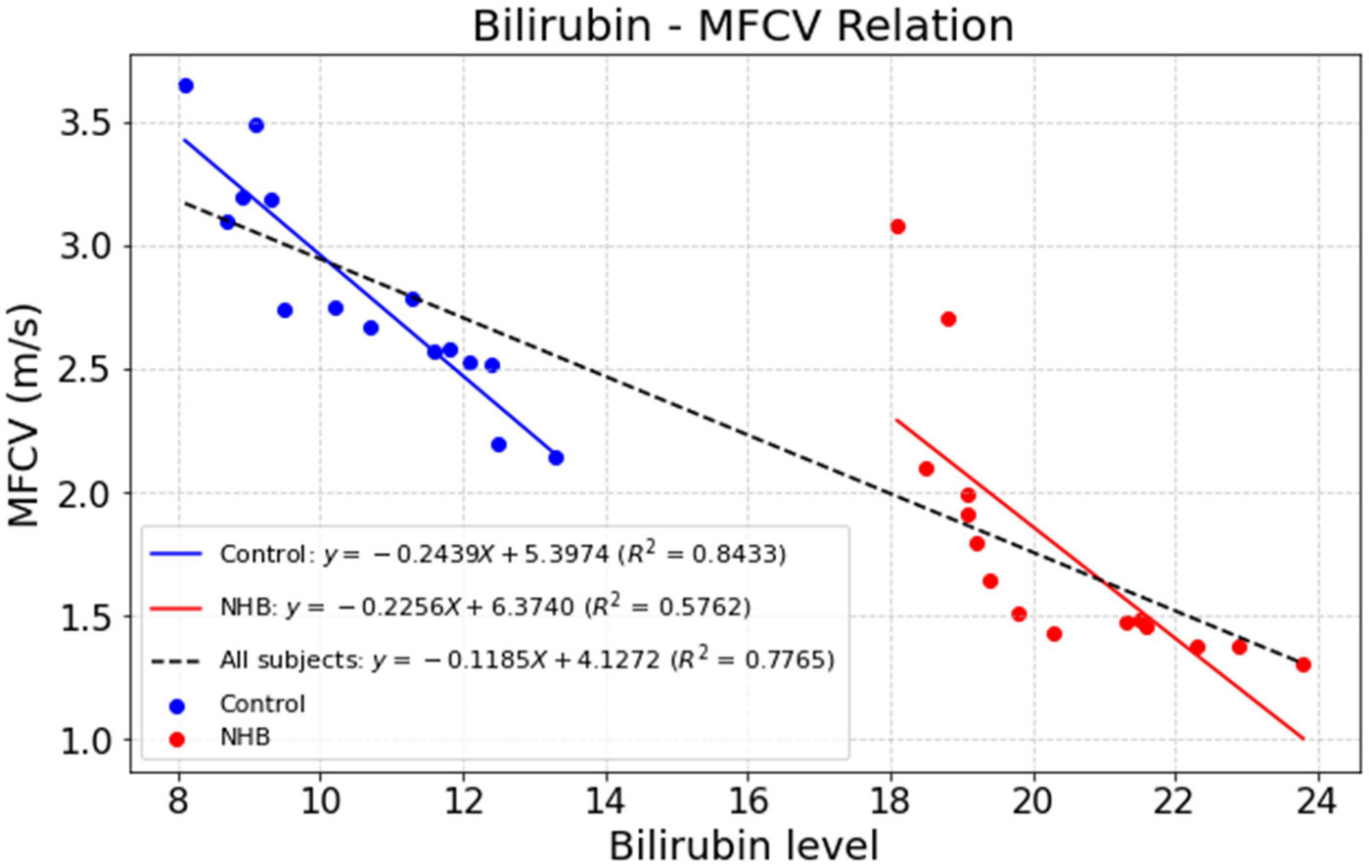
Linear regression of MFCV against bilirubin levels in neonates. A consistent negative association was observed across NHB and control groups (*R*^2^ = 0.576–0.843), with an overall slope of −0.119 (*p* < 0.001).

Linear regression analysis revealed a robust linear relationship between MFCV and bilirubin levels (*R*^2^ = 0.777). As shown in Figure 2, both NHB subjects and control neonates exhibited similar goodness-of-fit, with R² values ranging from 0.576 to 0.843 across the NHB group, control group, and the entire cohort, indicating a consistent linear association. Two-sample *t*-tests showed no significant differences between the NHB and control groups (*p* > 0.05), suggesting that group classification did not significantly affect the MFCV–bilirubin relationship. Across all neonatal subjects, higher bilirubin levels were associated with lower MFCV values, indicating a progressive decline in MFCV with increasing bilirubin concentration. Specifically, the slope of this linear relationship was −0.119, which was significantly different from zero as confirmed by a one-sample t-test (*p* < 0.001).

**Figure 3:**
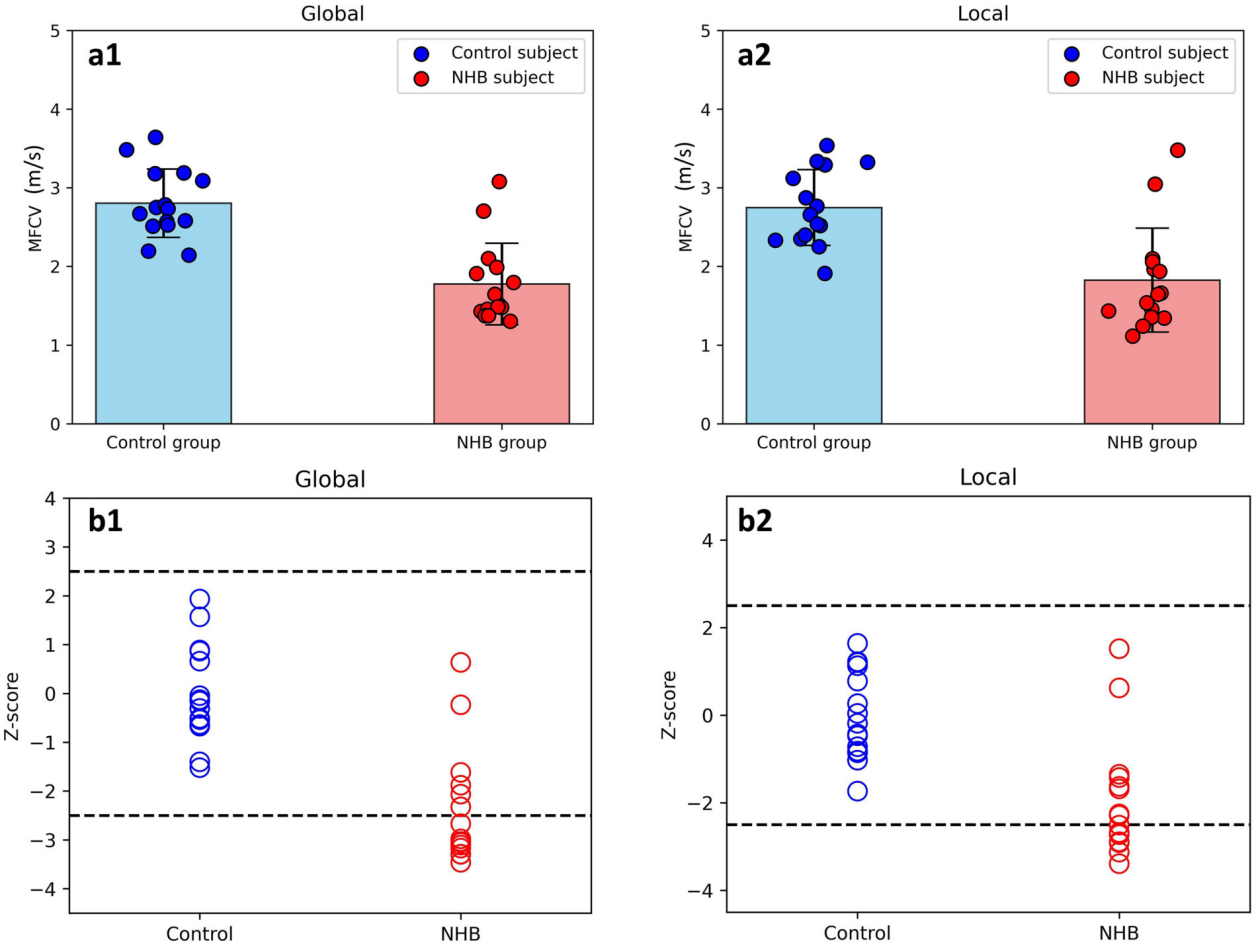
Group comparisons of MFCV **(a)** and Z-score **(b)** under the global (1) and local (2) schemes. NHB neonates showed significantly lower MFCV and abnormal Z-score values compared to controls under both schemes.

Figure 3 illustrates the average MFCV values (Figure 3a) and corresponding Z-scores (Figure 3b) under two time-delay schemes (global and local) for both groups. Under both schemes, the NHB group showed significantly slower MFCV than controls [global (a1): NHB: 1.776 ± 0.518 m/s, control: 2.804 ± 0.434 m/s, *p* < 0.001; local (a2): NHB: 1.825 ± 0.661 m/s, control: 2.748 ± 0.482 m/s, *p* < 0.001]. Z-score analysis revealed abnormal distributions of MFCV in the NHB group, with values ranging from −3.296 to 0.635 for global estimation (b1) and −3.392 to 1.516 for local estimation (b2). In contrast, all Z-score values in the control group remained within the normal range (±2.5). Significant between-group differences were observed in mean Z-scores for both global (*F* = 34.738, *p* < 0.001) and local estimations (*F* = 19.107, *p* < 0.001).

**Figure 4:**
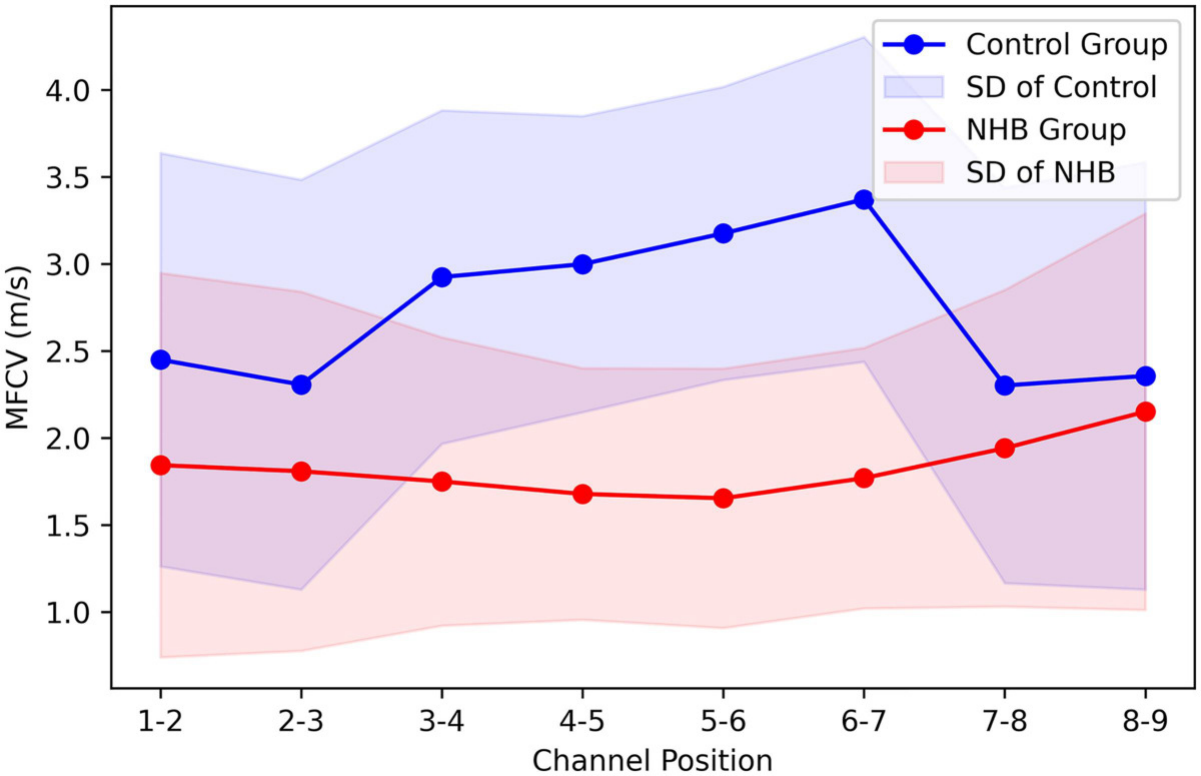
MFCV profiles across electrode channels in representative healthy and NHB neonates. Healthy neonates showed pronounced channel-wise fluctuations, whereas NHB neonates exhibited flatter distributions, indicating reduced spatial variation in MU activation. Shaded areas represent standard deviation across trials.

To further investigate signal propagation characteristics, MFCV values across each electrode interval were calculated using the local strategy, leveraging the linear electrode array's capability to capture conduction trends. This analysis revealed the influence of propagation patterns on MFCV in NHB subjects. Figure 4 presents the average MFCV values across all subjects for each of the 8 electrode intervals. In the control group, a clear “arch-shaped” distribution was observed, with relatively lower MFCV values at the proximal (IZ) and distal (tendon) intervals, and higher values at the middle electrode intervals. In contrast, the NHB group showed minimal variation across intervals, forming an almost flat profile. Linear regression yielded an average slope of −0.012, and a one-sample t-test indicated no significant deviation from zero (*p* > 0.05).

**Figure 5:**
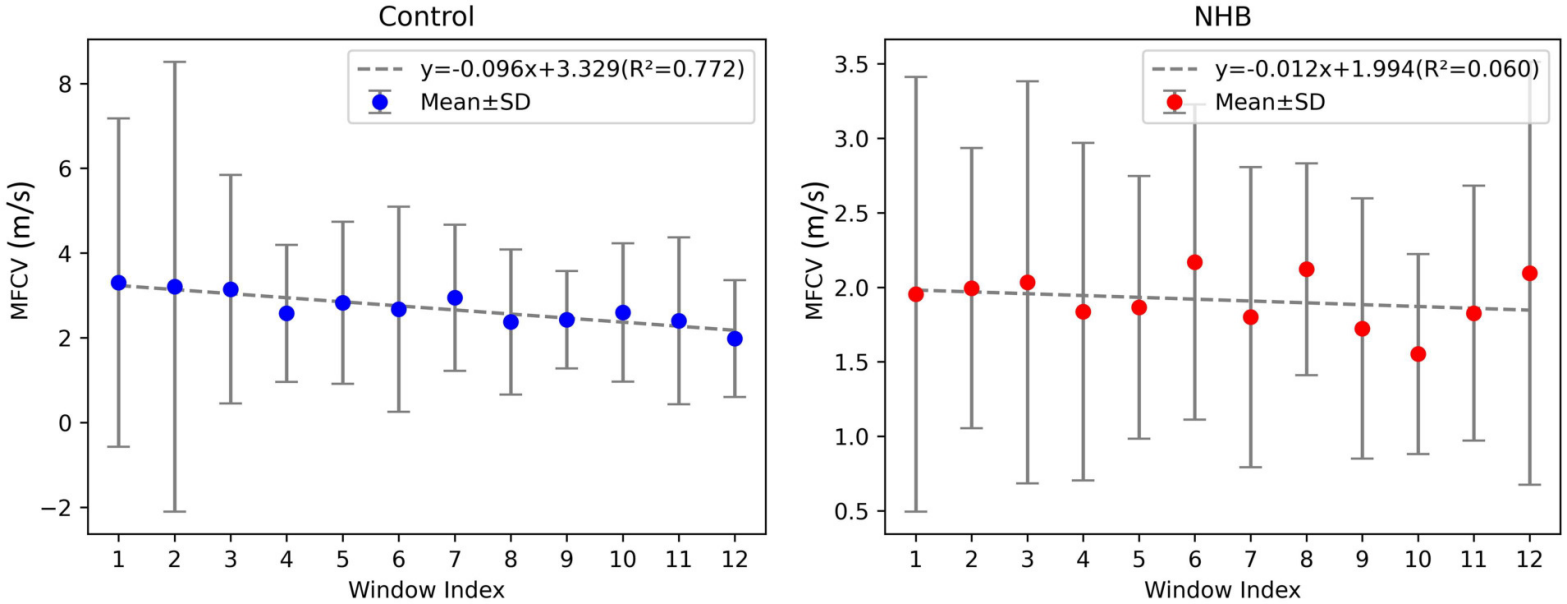
Temporal changes in MFCV during muscle contraction. Control neonates showed a significant decline in MFCV over time, while NHB neonates lacked this trend, indicating impaired neuromuscular responsiveness. Error bars represent standard deviation.

Figure 5 reports the MFCV values across multiple consecutive time windows within the 3-second contraction period using two estimation strategies. Linear regression analysis of the MFCV–time relationship revealed a robust linear correlation in all control subjects, with R² values ranging from 0.601 to 0.897 (mean ± SD: 0.772 ± 0.103) and an average slope of −0.096. In contrast, no significant linear relationship was observed in the NHB group, with R² values ranging from 0.002 to 0.203 (mean ± SD: 0.060 ± 0.062) and an average slope of −0.012. Although both groups showed a general trend of negative correlation between MFCV and time, the individual slope values did not differ significantly from zero in either group (*p* > 0.05).

**Figure 6:**
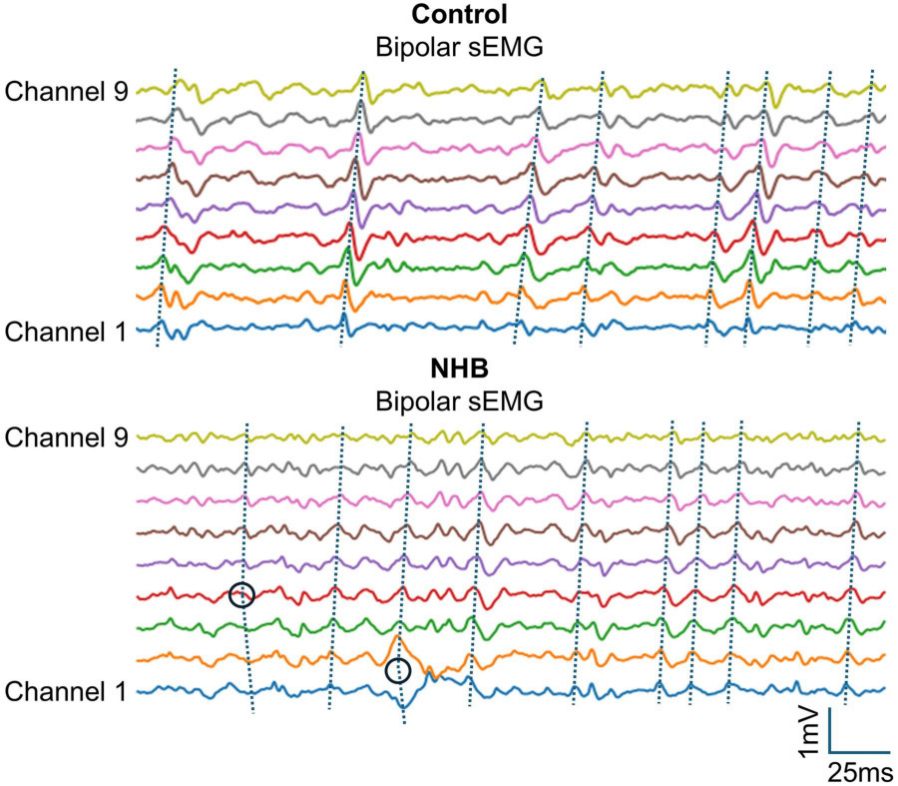
Illustration of IZ distribution in representative control and NHB subjects. The IZs of the Control subject was localized to a single channel, with MUAPs propagating unidirectionally toward the distal tendon. In contrast, the NHB subject showed IZs spanning channel 1–2 interval and channel 4, along with scattered MUAPs exhibiting polarity reversals. The sEMG signal length was 0.2 s.

To further illustrate abnormalities in innervation zone (IZ) distribution, representative subjects from both the control and NHB groups were selected, and sEMG signals from all 9 channels of the electrode array were plotted in Figure 6. In the control subject, the IZ was localized within a single channel, and the MUAPs propagated clearly in one direction from the IZ toward the distal tendon. In contrast, the NHB subject exhibited IZs distributed across multiple channels, along with scattered MUAPs showing polarity reversals.

## 4 Discussion

This study investigated the potential of MFCV as a biomarker to detect neuromuscular developmental abnormalities in newborns with NHB. The main findings are as follows (1): A linear relationship between MFCV and bilirubin levels was observed in both NHB and control subjects, with MFCV decreasing as bilirubin level increased; (2) MFCV values were significantly lower in NHB subjects compared to healthy controls, indicating impaired neuromuscular development; (3) NHB subjects exhibited abnormal spatiotemporal patterns of MFCV, including both inter-channel variation and deviations in the MFCV–time relationship; (4) The distribution of innervation zones in NHB subjects was more irregular than in healthy subjects.

Linear regression analysis confirmed a strong correlation between MFCV and bilirubin levels, with comparable R² values observed in both NHB and control groups (Figure 2), indicating a consistent sensitivity of MFCV to varying bilirubin concentrations. These findings are in line with previous reports indicating that elevated bilirubin levels can impair neuromuscular conduction and muscle fiber excitability (3, 24). The observed decrease in MFCV with increasing bilirubin may reflect bilirubin-induced alterations in the electrical properties of motor units or neuromuscular junctions. Importantly, the consistency of the linear relationship across NHB and control neonates suggests that MFCV may serve as a sensitive electrophysiological marker for monitoring bilirubin-related neuromuscular effects in neonates, independent of clinical classification.

The MFCV and the Z-score values in the NHB group were significantly lower than those in health controls (Figure 3, *p* < 0.05). These findings suggest that MFCV may serve as a valuable electrophysiological marker of abnormal neuromuscular development in neonates with hyperbilirubinemia. Specifically, MFCV is a sensitive indicator of muscle fiber membrane potential and ion permeability (19, 23, 24). Previous studies in healthy adults have reported typical MFCV values ranging from 3 to 6 m/s (24). In this study, the MFCV values of all healthy neonates—regardless of the time-delay estimation scheme used—ranged between 1.306 and 3.643 m/s, consistent with prior findings that conduction velocities in neonatal peripheral nerves are generally lower than those in adults (14).

In contrast, the NHB group exhibited significantly lower average MFCV values. This may reflect delayed neural development, reduced myelination, and impaired ion channel function in NHB neonates, resulting in slower propagation of MUAPs (2, 31, 32). The corresponding Z-score distributions supported this finding, as more than 7 NHB neonates fell below the normal range. This could be attributed to reduced MU recruitment, decreased MU firing rates, increased MU synchronization, and compressed recruitment thresholds, resulting in the activation of fewer and smaller MUs and ultimately reduced MFCV (33, 34). These results align with prior studies that even neonates without clinically apparent acute bilirubin encephalopathy may still be at risk for long-term neurodevelopmental impairment (2, 6, 13).

Interestingly, several NHB neonates displayed Z-score values within the normal range (±2.5), suggesting that their neuromuscular development may have remained largely intact. In such cases, phototherapy or exchange transfusion may have effectively reduced bilirubin levels early enough to prevent damage to neuromuscular junctions (1, 5). These neonates may have retained larger or more functional MUs, leading to higher MFCV values. Collectively, the heterogeneity in MFCV among NHB neonates underscores variability in neuromuscular development outcomes, potentially linked to the timing or adequacy of phototherapy cessation (4–6).

Moreover, we observed pronounced fluctuations in MFCV across the linear electrode array in healthy neonates, suggesting that MFCV estimation is highly sensitive to electrode position (Figure 4). These variations likely reflect differences in muscle tissue content beneath the electrodes. Previous research indicates that MFCV increases with muscle fiber diameter and that both the innervation zone (IZ) and tendon regions contain fewer muscle fibers and smaller MUAPs, often with polarity reversals. This may lead to systematic overestimation of MFCV in these regions (19, 20, 35). Therefore, we recommend visually identifying the IZ and tendon locations prior to electrode placement and positioning the array between these zones to improve reliability. By contrast, the NHB group exhibited less MFCV variation across electrode channels. This may reflect reduced MU activity and atypical neuromuscular maturation, resulting in similar MU sizes and distributions near both the IZ and tendon regions (10, 29, 36–38). These patterns further support the notion of impaired neuromuscular development in the NHB group.

Additionally, MFCV exhibited temporal dynamics (Figure 5). In control subjects, a significant negative linear correlation between MFCV and contraction time was observed, consistent with prior reports (23, 39, 40). This decline is likely due to reduced membrane excitability caused by extracellular potassium accumulation and metabolic acidosis (e.g., lactate buildup), both of which slow action potential propagation (23, 41). However, this temporal relationship was absent in the NHB group, suggesting a diminished neuromuscular response to sustained contraction. This may reflect bilirubin-induced alterations in peripheral nerve excitability, synaptic transmission, or muscle membrane properties, as shown in previous *in vitro* and animal studies (14, 34, 42). These findings imply that hyperbilirubinemia may disrupt both central and peripheral neuromuscular development, leading to atypical conduction dynamics during early infancy (3, 32, 34).

The use of a linear electrode array also facilitated visualization of IZ locations (Figure 6). In healthy neonates, IZs were typically localized to a single channel, indicating spatially organized MUAP propagation. In contrast, IZs in the NHB group were more dispersed, spanning multiple channels. This irregular pattern may reflect delayed myelination and abnormal neuromuscular junction formation (33, 37, 38). Such IZ disorganization has previously been associated with immature or damaged neuromuscular systems, potentially due to reduced MU synchronization (34, 42–44). In the context of hyperbilirubinemia, these findings support the notion that elevated bilirubin levels may interfere with the early postnatal establishment of stable neuromuscular architecture.

This study has several limitations. Due to the relatively long follow-up period, a portion of enrolled neonates were lost to follow-up, resulting in a modest sample size (*n* = 15), which limited the ability to perform subgroup-level statistical analyses within the NHB group. Additionally, this study relied on linear sEMG arrays for MFCV estimation but did not perform motor unit decomposition. Future work will employ HD-sEMG to investigate motor unit activity patterns in NHB neonates in greater detail.

## Data Availability

The raw data supporting the conclusions of this article will be made available by the authors, without undue reservation.
